# Glomerular Hematuria: Cause or Consequence of Renal Inflammation?

**DOI:** 10.3390/ijms20092205

**Published:** 2019-05-05

**Authors:** Juan Antonio Moreno, Ángel Sevillano, Eduardo Gutiérrez, Melania Guerrero-Hue, Cristina Vázquez-Carballo, Claudia Yuste, Carmen Herencia, Cristina García-Caballero, Manuel Praga, Jesús Egido

**Affiliations:** 1 Renal, Vascular and Diabetes Research Laboratory. Fundacion Jimenez Diaz University Hospital-Health Research Institute (FIIS-FJD), Autonoma University of Madrid (UAM), 28040 Madrid, Spain; mel10anie@gmail.com (M.G.-H.); cvazqu01@ucm.es (C.V.-C.); carmen_herencia@hotmail.com (C.H.); crisgcomplutense@gmail.com (C.G.-C.); 2 Department of Cell Biology, Physiology, and Immunology, Maimonides Biomedical Research Institute of Cordoba (IMIBIC), University of Cordoba, 14014 Cordoba, Spain; 3 Department of Nephrology, Hospital 12 de Octubre, 28040 Madrid, Spain; sevillano.am@gmail.com (Á.S.); eduardogutmat90@hotmail.com (E.G.); claudiayustelozano@yahoo.es (C.Y.); mpragat@senefro.org (M.P.); 4 Spanish Biomedical Research Centre in Diabetes and Associated Metabolic Disorders (CIBERDEM), 28040 Madrid, Spain

**Keywords:** hematuria, inflammation, oxidative stress, tubular injury, AKI, chronic kidney disease (CKD)

## Abstract

Glomerular hematuria is a cardinal symptom of renal disease. Glomerular hematuria may be classified as microhematuria or macrohematuria according to the number of red blood cells in urine. Recent evidence suggests a pathological role of persistent glomerular microhematuria in the progression of renal disease. Moreover, gross hematuria, or macrohematuria, promotes acute kidney injury (AKI), with subsequent impairment of renal function in a high proportion of patients. In this pathological context, hemoglobin, heme, or iron released from red blood cells in the urinary space may cause direct tubular cell injury, oxidative stress, pro-inflammatory cytokine production, and further monocyte/macrophage recruitment. The aim of this manuscript is to review the role of glomerular hematuria in kidney injury, the role of inflammation as cause and consequence of glomerular hematuria, and to discuss novel therapies to combat hematuria.

## 1. Introduction

Hematuria is described as the presence of red blood cells (RBCs) in the urine. Dysmorphic (abnormally shaped) RBCs in the urine are the consequence of RBC egression through the glomerular filtration barrier, and indicate hematuria of glomerular origin. Glomerular hematuria is a frequent manifestation of many renal diseases, and may be classified as microscopic or macroscopic according to its intensity. Recent evidence suggests a negative repercussion of glomerular hematuria on kidney function. In addition, gross hematuria promotes acute kidney injury (AKI), with a subsequent impairment of renal function by different pathological mechanisms, including an exacerbated inflammatory response. In the next sections, we will fully address the role of glomerular hematuria on kidney injury, emphasizing the causes as well as the pathophysiological consequences.

## 2. Glomerular Hematuria: An Important and Often-Neglected Clinical Sign

### 2.1. Prevalence of Glomerular Hematuria

The prevalence of hematuria in the general population is certainly unsettled. Screening programs show hematuria in 0.18–16.1% of healthy adults [1,2,3,4,5] and in between 0.03% and 3.9% of children [6,7,8,9]. This broad range reflects a relative lack of interest in hematuria, based in its traditionally benign consideration, the absence of standardized methods to detect and quantify hematuria [10,11], or even the difficulties in distinguishing non-glomerular from glomerular hematuria. Moreover, there is striking limited data on the prevalence and severity of glomerular hematuria in renal biopsy registries [12,13,14,15,16]. In these studies, the occurrence of glomerular hematuria ranged between 63.7% and 75.8% of cases. Glomerular hematuria seems to be more frequent in males than in females, regardless of age [17]. Children present more frequent macroscopic hematuria bouts than adults, whereas microhematuria is more common in adults [12,17].

### 2.2. Common Causes of Glomerular Hematuria

IgA nephropathy (IgAN), the commonest primary glomerulonephritis (GN), is the most frequent cause of glomerular hematuria (Table 1) [12,17]. Approximately half of patients can present with outbreaks of macroscopic gross hematuria (MGH), while the other half can do so with microhematuria. Macroscopic bouts of hematuria are more common in the early stages of IgAN and in children, concomitant with mucosal infections, usually in the respiratory tract and occasionally in the gastrointestinal tract [18]. 

Rapidly progressive glomerulonephritis (RPGN), vasculitis, and acute glomerular inflammation, as observed in postinfectious GN or lupus, may also be associated with glomerular hematuria. Although hematuria is a usual urinalysis feature in endocapillary GN [23], extracapillary GN [12], and membranoproliferative GN [24], the real prevalence of hematuria in these diseases is mainly based in observational cohorts [25,26,27]. Data from the Spanish renal biopsy registry [17] reported an unexpectedly high rate of hematuria (50%) among patients that were traditionally considered as not hematuric GN, including minimal change disease, membranous GN, or focal and segmental glomerulosclerosis. In the recently characterized C3 glomerulonephritis (C3GN), hematuria—mainly microhematuria—was also present in 87% of the patients [28]. Interestingly, macrohematuria bouts have been also described in C3GN concurrently with upper respiratory tract infections, mimicking IgAN [29].

Current advances in genetic testing have allowed the condition previously known ”benign familial hematuria“ to be split into several type-IV collagen-associated diseases, such as Alport syndrome, thin basement membrane disease (TBMN), and the hereditary angiopathy, nephropathy, aneurysms and muscle cramps syndrome (HANAC) [30]. These type IV collagen-related disorders show persistent microscopic hematuria in the early stages, which progress over the years to proteinuria and chronic kidney disease (CKD), dependent on individual genetic background [31]. Further, infections have been reported as a trigger of macroscopic bouts of hematuria in collagen-associated disorders [32,33]. Finally, anticoagulant-related nephropathy (ARN) is a recently described entity characterized by gross glomerular hematuria and AKI in patients receiving warfarin [34] or other types of anticoagulant therapy [35,36]. ARN is secondary to a profuse glomerular hemorrhage as a consequence of over-anticoagulation (INR > 3) [37]. ARN shows a disease rate of 2–26 cases per year of follow-up [38,39,40] and a prevalence of the entity of 20% in over-coagulated patients (INR > 3) [41]. Unpublished data from our group shows that anticoagulant therapy is associated with AKI related to hematuria bouts in patients with IgAN. This could be a link between ARN and IgAN. 

## 3. Hematuria and Renal Damage: Cause or Consequence?

### 3.1. Hematuria as a Sign of Glomerular Inflammation and Disease Progression

Although hematuria is a cardinal symptom of renal disease, it has occupied very little relevance as a negative prognostic factor, unlike proteinuria that continues to play a central role in the diagnosis and treatment of kidney diseases. This lack of interest in hematuria is surprising because it is a defining symptom of IgAN and other pathologies. New evidence suggests a link between inflammation and the genesis of glomerular hematuria. In an experimental model of IgAN, treatment with an IgA1 protease decreased IgA1 deposition—a fact that correlated with a decrease in inflammation, mesangial expansion, and from a clinical point of view, with a very significant reduction in hematuria without a significant influence on proteinuria [42]. In IgAN patients, macroscopic hematuria coincides with aero-digestive infections [43], indicating that dysregulation of the mucosal immune system may play an important role in the pathogenesis of hematuria through a mucosa–kidney axis [44]. In fact, the activation of Toll-like receptors (TLRs) by bacterial or viral antigens causes polyclonal lymphocyte proliferation [45,46] and the formation of circulating immune complex [43,47]. Another possible mechanism involved in the exacerbation of macrohematuria by inflammation includes the CX3CL1/CX3CR1 axis. The peripheral mononuclear cells of patients with IgAN have a higher expression of CX3CR1 during the episode of macrohematuria, as well as increased serum and urinary levels of CX3CL1, the unique ligand of CX3CR1 [48]. The CX3CL1/CX3CR1 axis may play a primordial role through its chemotactic activity to recruit infiltrating cells that can modify the glomerular filtration barrier. Increased CXC3CL1 expression is not exclusive to IgAN, and can be increased in other glomerular diseases associated with hematuria, such as lupus nephritis [49].

Recent data have shown the value of hematuria as a possible marker of relapse in antineutrophil cytoplasmic antibody (ANCA)-associated vasculitis. The authors found a significant positive association between the persistence of hematuria and subsequent nephritis relapse, although this association was not significant with the persistence of proteinuria [20]. One of the great future challenges is the establishment of a clear association between the glomerular damage and its potential utility to decide if it is necessary to increase the immunosuppressive treatment. Following these studies, the question arises as to whether hematuria is a simple marker of major renal damage. Hematuria could be an early indicator of recurrence, particularly if we consider that this is the first study examining the value of the level of hematuria in the relapse of vasculitis. Hematuria was previously demonstrated as a marker of flare in patients with systematic lupus erythematosus (SLE). Ding et al. suggested that alterations in the sediment, either in the form of isolated microhematuria or associated with sterile pyuria, were associated with the activity of the SLE, being able to serve as a marker of activity phase [19]. This hypothesis was confirmed in an analysis based on a prospective study of urine sediment changes in the Ohio SLE study [50].

### 3.2. Pathophysiological Consequences of Hematuria (AKI and CKD)

#### 3.2.1. Hematuria and AKI

Renal findings in pathologies related to glomerular hematuria usually include tubules filled with RBC casts and acute tubular necrosis, mainly during the gross hematuria bouts. Massive hematuria of glomerular origin produces AKI and damage in tubular cells through different mechanisms: (a) direct tubular damage due to intratubular obstruction of the blood casts, (b) direct tubular toxic effect of hemoglobin (Hb) and heme produced after rupture of the erythrocytes in the tubular lumen, and (c) processes of erythrophagocytosis by the renal tubular cells. Taking all these aspects into account, the duration of macrohematuria bouts becomes a crucial phenomenon for the recovery of renal function. This may be especially relevant in elderly patients and patients with previous chronic renal failure, who may not be able to recover their full functional capacity, especially if the insult is prolonged [21,51].

**Figure 1:**
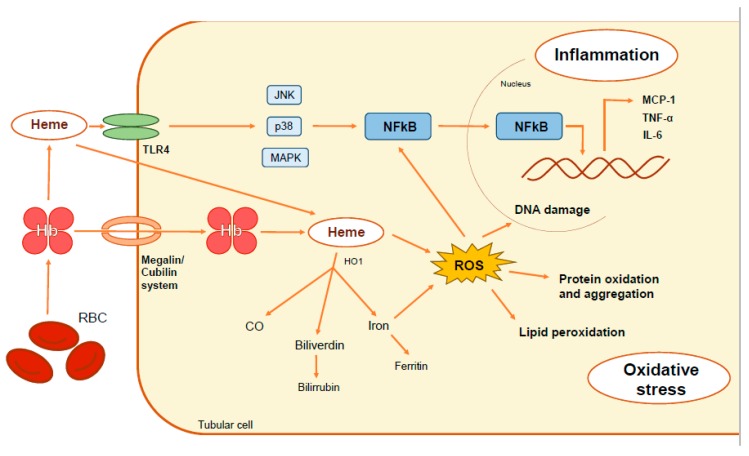
Pathophysiological mechanisms involved in renal damage associated with hematuria. Hemoglobin released by intratubular degradation of erythrocytes may be incorporated into proximal tubules through the megalin-cubilin receptor system or degraded in the tubular lumen, releasing heme. Hb, heme and iron accumulation within tubular cells triggers oxidative stress (lipid peroxidation, protein oxidation and aggregation and DNA damage) and inflammatory cytokine secretion (MCP-1 (monocyte chemoattractant protein 1), TNF-alpha (tumor necrosis factor-alpha), and interleukin 6 (IL-6)) throughout NF-κB transcription factor activation. The heme group may be recognized by the Toll-Like Receptor 4 (TLR4), resulting in the activation of the pro-inflammatory downstream signaling pathways like c-Jun kinases, p38, MAPK and NF-κB.

From a physiopathological point of view, red blood cells in the urine in patients with hematuria release Hb and heme-related products, which are further taken up by tubular cells (Figure 1). Once inside the cell, Hb dissociates, releasing the globins and the heme group, which induces several pathological effects, including oxidative stress, cell death, inflammation, and fibrosis [51,52]. Recent data from our group show that, in addition to the tubular cells, podocytes may be the cellular target of Hb-mediated kidney damage. Thus, Hb induces oxidative damage, podocyte dysfunction, and finally apoptosis, with the detachment of the podocyte from the glomerular capillary [53]. The traffic of Hb through the capillary wall can damage the podocyte, and consequently originate an alteration of the glomerular filtration barrier in patients with glomerular diseases with outbreaks of macroscopic hematuria, such as IgAN. This could explain, at least in part, the deterioration of renal function suffered by those patients after a hematuria bout, especially in elderly patients [21]. However, this hypothesis has not yet been tested. The injury suffered by erythrocytes during their pass throughout the glomerular filtration barrier may promote the release of microvesicles containing microRNA (miRNA). miRNA can be swallowed by nearly all cells, playing an important role in the regulation of oxidative stress and intercellular communication by regulating gene expression. The more prevalent miRNA present in the urinary sediment of IgAN patients with hematuria were mainly derived from urinary erythrocytes, such as miR-25-3p, miR-144-3p, and miR-486-5p [54]. That implies that miRNA delivered by hematuria could act over renal parenchymal cells, changing their gene expression, and could be involved in the pathogenesis and evolution of kidney disease [55]. In other words, the presence of specific miRNA in the sediment could be a marker of the activity of hematuric disease, and may be a useful diagnostic tool. Thus, miR-215-5p and miR-378i appear more frequently in IgAN patients [54,56]. 

#### 3.2.2. Hematuria as a Risk Factor for CKD

For many years, hematuria has been considered as only a symptom of some renal disorders. However, multiple studies have challenged this concept, pointing out that the presence of hematuria is associated with an increased risk of developing end-stage renal disease (ESRD). Glomerular diseases, specially IgAN, constitute the scenario where the association between hematuria and long-term renal dysfunction have been best analyzed. In this way, the persistence of hematuria in IgAN has been related to a greater probability of developing ESRD compared to patients with minimal or negative hematuria [22]. In fact, in that disease mild hematuria is associated with an increased risk of ESRD after 10 years of follow-up [57].

In an epidemiological study, the presence of isolated microhematuria significantly increased the risk for ESRD after 22 years of follow-up in a young Israeli population [6]. The presence of hematuria was also associated with a faster decline in renal function in advanced CKD patients compared with those without hematuria [58]. Similar findings emerged from the Chronic Renal Insufficiency Cohort (CRIC) Study [59] and the EPPIC (Evaluating Prevention of Progression In Chronic kidney disease) trials [60]. These studies evidenced an increase of the risk of ESRD after two years of follow-up among patients with baseline hematuria and a decrease of the risk to develop ESRD in those without hematuria. Finally, a recent report showed a significant association of hematuria with an increased risk of ESDR in a group of patients with diabetic nephropathy—the first cause of CKD in developed countries [61]. Based on these pieces of evidence, we consider that hematuria should be added to the traditional risk factor of CKD progression.

## 4. Should Hematuria be Included as a Surrogate Marker in Clinical Trials of Renal Diseases?

The search for adequate surrogate markers of disease progression, including the progression of renal diseases, is a key issue in many clinical conditions. However, the doubling of serum creatinine is currently the only validated marker associated with renal outcome [62]. Some evidence indicates that proteinuria disappearance could also be a good candidate as a surrogate marker, but further studies are necessary before its acceptance [62]. Hematuria is considered a marker of activity in ANCA vasculitis, lupus nephritis, or IgAN [19,20,63], and recent data indicate that the persistence of hematuria in IgAN is associated with increased risk of the development of ESRD [21,64,65]. For that reason, hematuria disappearance could be a surrogate marker of renal outcome in different diseases, such as vasculitis or glomerulonephritis. However, additional studies are necessary to validate this hypothesis.

## 5. Hematuria at the Crossroads of Inflammation and Oxidative Stress

The lysis of RBC in the urinary space releases Hb and heme-related products. Hb is incorporated into the kidney through the megalin/cubilin system, and after their oxidation and intracellular destabilization, heme is released and exerts its cytotoxic effect on the renal cells (particularly on the renal tubular epithelium) [66]. Heme consists of a tetrapyrrole ring with an iron atom bound in the center, coordinated to four pyrrole rings [67]. Heme released from the RBC generates oxidative stress, promoting the oxidation of proteins and lipids [68,69], altering the integrity of the cells, and damaging the DNA [70]. The structural instability of heme is essential for the induction of oxidative stress and inflammatory response [71]. Because of its structural properties, especially the hydrophobicity of the porphyrin ring, heme can be incorporated in the lipid bilayer that constitutes cell membranes, where it increases cellular susceptibility to oxidative damage [72]. Additionally, free iron is a potent oxidant and can generate free radicals through the Fenton reaction.

Heme has proinflammatory properties, including leukocyte activation and migration, increased expression of adhesion molecules, and the induction of acute-phase proteins. Thus, heme promotes endothelial activation and elicits the expression of adhesion molecules such as ICAM-1, VCAM-1, E-selectin, P-selectin, and von Willebrand factor [73,74,75], which facilitates the recruitment and migration of leukocytes [73,76]. 

Heme also promotes direct pro-inflammatory effects, for example, by activating the production of leukotriene B4 in tissue macrophages, which acts as a chemotactic agent of neutrophils [77]. Additionally, heme can act directly on neutrophils by delaying their apoptosis [78], enhancing their harmful effect on tissue [78]. Heme has been identified as a damage-associated molecular pattern (DAMP), a group of endogenous molecules derived from damaged cells capable of promoting and exacerbating the immune response. These DAMPs are recognized by pattern-recognition receptors (PRRs), within the family of TLRs. Specifically, heme is a ligand of a member of this family—the TLR4 receptor [79]. The activation of TLR4 by heme induces an inflammatory response through the activation of the transcription factor NF-kB [75,80]. As a ligand of TLR4, heme promotes the activation of several downstream signaling pathways, such as c-Jun kinases, p38, and MAPK [81], thus inducing an inflammatory response throughout MCP-1 production [82]. The TLR4 antagonist TAK-242 reduced these heme side effects, suggesting a direct relationship between inflammation and heme through TLR4 in renal tubular cells. Another pathway involved in the inflammation induced by heme is the activation of the NLRP3 (nitrogen permease regulator-like 3) inflammasome, leading to the release of different cytokines and chemokines involved in the recruitment of monocytes/macrophages [83].

## 6. Can We Envision a Therapeutic Approach for Hematuria-Associated Diseases?

There are no specific therapies to decrease adverse effects associated with hematuria in glomerular diseases. However, the more plausible therapeutic option would be oriented towards the prevention of hematuria bouts and treatment of the nephrotoxic effect of hematuria on renal cells.

### 6.1. Treatment for the Prevention of Gross Hematuria Bouts

Since hematuria was considered as just a benign symptom for decades, no significant effort has been made to treat macrohematuria. Indeed, the Kidney Disease: Improving Global Outcomes (KDIGO) guidelines only recommend supportive treatment for IgAN patients in this context, although this population is at high risk of developing acute or chronic kidney failure [21]. Immunosuppressor therapy, and most specifically corticosteroids, seems a logical option to treat macrohematuria episodes because inflammation is implicated in this phenomenon and because patients with IgAN, ANCA-vasculitis, or lupus nephritis that respond to this therapy usually experience hematuria disappearance. However, to date, there is no evidence to support this hypothesis. 

The introduction of budesonide—a new oral corticosteroid with fewer systemic adverse events—provides opportunities to further explore therapeutic approaches for patients with persistent episodes of macroscopic hematuria [84]. Interestingly, budesonide treatment reduced hematuria in IgAN patients from the NEFIGAN clinical trial [85]. Since macrohematuria bouts are usually preceded by upper respiratory infection, tonsillectomy may be considered as another approach in IgAN patients with recurrent tonsillitis, though the results are contradictory [86].

### 6.2. Treatment of the Nephrotoxic Effect of Hematuria

No drug therapy has yet been tested in the management of nephrotoxicity induced by glomerular hematuria. However, based on the actual knowledge of pathogenesis of the damage, some therapeutic options could be effective: (1) alkalinization of the urine to reduce the dissociation of iron from the Hb, (2) diuretics to tubular obstruction by RBC casts, (3) iron chelators (deferiprone or deferoxamine) to decrease iron-nephrotoxicity, and (4) anti-oxidant and anti-inflammatory drugs, such as N-acetylcysteine or Nrf2 inducers. Although there are no current studies reporting beneficial effects on hematuria, a recent paper showed that the administration of N-acetylcysteine prevented Scr increase in 5/6 nephrectomized rats with warfarin-induced AKI [87]. 

## 7. Conclusions

Hematuria is a common urinary finding present in several glomerular diseases and patients with bad control of coagulation, among other conditions. Recent studies indicate a pathological role of hematuria in renal damage by promoting AKI and progression to CKD. The renal damage mediated by hematuria is related to nephrotoxic actions of Hb and heme on tubular cells, although data from our studies may suggest that the podocyte may be another cellular target of these molecules. Inflammation and oxidative stress are key processes involved in hematuria-related diseases, and may be plausible targets to develop novel therapeutic approaches.

## Figures and Tables

**Table 1:** Significance of hematuria in glomerular disease.

Disease	Significance
Lupus nephritis	Classical symptom Marker of activity [19]
ANCA-associated vasculitis	Classical symptom Marker of activity Marker of risk to relapse after response to therapy [20]
Disorders of collagen IV α345	Classical symptom
IgAN	Classical symptom Marker of activity Probable implicated in progression of the disease Implicated in AKI associated to gross hematuria [21] Probable risk factor to progression to ESRD [22]
Other primary glomerulopathies	Classical symptom Marker of activity [19]
